# Development and protective efficacy of multi-epitope vaccine FL46 against cystic echinococcosis

**DOI:** 10.3389/fimmu.2025.1686959

**Published:** 2025-10-16

**Authors:** Tingting Jiang, Maerdan Mahemuti, Weina Wang, Shuai Han, Xiaoying Wu, Hua Liu, Quan Chen, Xiaojin Mo, Xu Wang, Aerxin Kadiaili, Yuan Hu, Jianping Cao, Shizhu Li

**Affiliations:** ^1^ National Institute of Parasitic Diseases, Chinese Center for Disease Control and Prevention, Chinese Center for Tropical Diseases Research, National Key Laboratory of Intelligent Tracking and Forecasting for Infectious Diseases, World Health Organization Collaborating Centre for Tropical Diseases, Key Laboratory of Parasite and Vector Biology, National Health Commission, Shanghai, China; ^2^ Surgery of Friendship Hospital, Yili Kazak Autonomous Prefecture of Xinjiang, Yining, China; ^3^ Clinical Research Institute of Friendship Hospital, Yili Kazak Autonomous Prefecture of Xinjiang, Yining, China; ^4^ The School of Global Health, Chinese Center for Tropical Diseases Research, Shanghai Jiao Tong University School of Medicine, Shanghai, China

**Keywords:** cystic echinococcosis, multi-epitope vaccine, *Echinococcus granulosus*, HLA-bound T-cell epitopes, Th1/Th2 immune response

## Abstract

*Echinococcosis* is an important zoonotic parasitic disease caused by *Echinococcus* spp. Infection. Vaccines represent the most economical and effective means of preventing and controlling echinococcosis. This study aimed to construct a multi-epitope vaccine targeting *E. granulosus* and evaluate its immunogenicity and protective efficacy against cystic echinococcosis. We identified HLA-bound T-cell epitopes (P1, P2, P3) from the liver of echinococcosis patients using co-immunoprecipitation and incorporated them into the multi-epitope vaccine FL46. *In vitro* cytotoxicity assessment using BMDCs and U937 cells confirmed that FL46 concentrations below 500 µg/mL did not impair cell proliferation. Forty C57BL/6 mice were randomly divided into vaccine or control groups. The vaccine group received three subcutaneous immunizations (100 µg FL46/mouse, emulsified 1:1 with Freund’s adjuvant) at two-week intervals. Two weeks post-final immunization, all mice were challenged intraperitoneally with 2000 protoscoleces and sacrificed eight months post-infection. Vaccinated mice exhibited significantly elevated serum levels of IL-2, TNF-α, IL-5, IL-6, and Keratinocyte-derived cytokine (KC) after immunization three times. Splenic B1 and B2 lymphocyte proportions increased dramatically eight months after the third immunization. Significantly higher levels of IgM, IgG, and IgG2a were detected in the vaccine group eight weeks post-infection, persisting for at least eight months. The vaccine group demonstrated a significantly reduced cyst burden (number and weight) compared to the controls, corresponding to a 59.16% cyst suppression rate. The indicators of liver fibrosis were also significantly lower in vaccinated mice. These results demonstrate that the multi-epitope vaccine FL46 elicits a robust mixed Th1/Th2 immune response and confers significant protection against cystic echinococcosis, highlighting its potential as a candidate vaccine.

## 1 Introduction

Echinococcosis is an important zoonotic parasitic disease caused by *Echinococcus spp.* infection and is distributed worldwide, seriously threatening human health and the development of animal husbandry (1, 2). Echinococcosis is divided into cystic echinococcosis (CE) and alveolar echinococcosis (AE). Among them, cystic echinococcosis is the most common and has a relatively high incidence (3, 4). Approximately 50 million people worldwide are infected with cystic echinococcosis, resulting in over one million disability-adjusted life years (DALYs). It endangers human health and the development of animal husbandry. China is one of the countries with the highest incidence of CE, mainly prevalent in the western regions dominated by animal husbandry, such as Xinjiang, Qinghai, and Tibet, accounting for 40% of the global disease burden (5–7).

*E. granulosus* mainly parasitizes the host′s organs, such as the liver, lungs, and spleen, causing harm to humans or animals through mechanical compression, allergic reactions, and toxin effects, among which the liver is the most severely affected organ (8, 9). The treatment of CE mainly includes surgery and drug therapy. Total cystectomy with CE significantly improves patients′ quality of life, but the effect is not ideal for multiple microcysts. Drug treatment currently relies on albendazole, but there is a lack of alternative drugs to albendazole, and new compounds are required urgently (10). The life cycle of AE and CE is complex. The adult parasitizes in the small intestine of canines (such as dogs and foxes). The larval stage (echinococcus) spreads to intermediate hosts (such as humans, cattle, and sheep) through the pollution of the environment by its eggs. Vaccines represent an essential preventive measure and the most economical and effective means of preventing and controlling CE (10). The Eg95 vaccine for sheep against CE has been launched on the market and has achieved a protective effect of over 90% (11). At the same time, vaccines for humans and dogs are still in the stage of laboratory research (12).

Echinococcosis vaccines include traditional vaccines, recombinant protein, nucleic acid-based, multi-epitope vaccines, each with advantages and limitations (13). Among them, multi-epitope vaccines have attracted much attention due to their benefits, such as flexible design, strong specificity, no risk of infection, and ease of production and storage (14). The selection and design of epitopes are the key to obtain high protective effects of the multi-epitope vaccine. The primary strategy is to predict T and B cell epitopes through immuno-informatics tools and molecular docking techniques or to identify antigenic epitopes bound to major histocompatibility complex (MHC) molecules by using liquid chromatography-mass spectrometry (LC-MS) technology (15–17). The determination of antigenic epitopes bound to MHC by LC-MS is more accurate and specific than the prediction of T and B cell epitopes by immuno-informatics tools. In this study, we adopted the co-immunoprecipitation technique to precipitate MHC-peptide from cell lysis buffer, separated polypeptide from the antibody-MHC-polypeptide complex, and identified the isolated peptide by mass spectrometry. The peptides of CE were screened and linked using the KKK and GPGPG linkers to form the multi-epitope vaccine FL46 to evaluate the immune response induced by FL46 and its protective effect against echinococcosis.

## 2 Materials and methods

### 2.1 The screening of epitopes and the synthesis of FL46

T-cell epitope peptide of *E. granulosus* was screened by Jingjie Biotechnology Co., LTD. The samples come from liver tissues surgically removed from echinococcosis patients. HLA-peptides were precipitated from the tissue lysis buffer using antibodies against HLA-I and HLA-II and separated from the antibody-HLA-polypeptide complex. The peptide sequences in the samples were detected by liquid chromatography-mass spectrometry. We obtained T cell epitopes (P1-P3) commonly in the samples. We used the KKK amino acid to link the P1 and P2 epitopes (binding to HLA-II), and the GPGPG amino acid to link the P2 and P3 epitopes (binding to HLA-I) to maintain the independence of each fragment. The multi-epitope vaccine FL46 was synthesized in Chinese Peptide Co., Ltd.

### 2.2 Safety evaluation

Bone Marrow-Derived Dendritic Cells (BMDCs) were collected from the tibia of mice and cultured for seven days. The cell concentrations were adjusted to 1×10^6^ cells/mL and cultured in 96-well plates. FL46 with different concentrations (20, 40, 80, 100, 200 μg/mL) was added to the cells, respectively, in the experiment group. After the cells were cultured for 24 h at 37 °C and 5%CO_2_, CCK8 reagent was added, and the cells were cultured for three h. The absorbance value at 450 nm was detected by an enzyme-linked immunosorbent assay (ELISA) reader, and the cell viability was calculated. The number of biological replicates was three.

After adjusting the density of U937 cells (the monocyte line) to 1×10^6^ cells/mL, U937 cells were inoculated in 24-well plates. Phorbol 12-Myristate 13-Acetate (PMA) was added at 100 ng/mL in each well, and cells were cultured for 48h to transform into macrophages. FL46 with different concentrations (200, 250, 300, 350, 400, 450, and 500 μg/mL) was added to the cells, respectively. Three duplicate wells were set for each concentration. The remaining operations were the same as those of BMDCs.

### 2.3 Animal immunization and infection

Forty 6-8-week-old C57BL/6 female mice were randomly divided into the vaccine and control groups. FL46 (100 μg/mouse) was mixed with an equal volume of complete Freund’s adjuvant (for the primary immunization) or incomplete Freund’s adjuvant (for the last two booster immunizations), respectively. C57BL/6 mice in the vaccine group received three subcutaneous immunizations (100 µg FL46/mouse, emulsified 1:1 with Freund’s adjuvant) at two-week intervals. Blood samples were collected from the orbital venous plexus of mice two weeks after each immunization. Two weeks post-final immunization, all mice were challenged intraperitoneally with 2000 protoscoleces and sacrificed eight months post-infection. We calculated the size, number, and weight of cysts in the abdominal cavity and detected the levels of cytokines and antibodies. All liver samples surgically removed from patients with echinococcosis and animal experiments were performed following the guidelines of the Laboratory of Animal Welfare and Ethics Committee (LAWEC) of China and approved by the LAWEC of the National Institute of Parasitic Diseases, Chinese Centre for Disease Control and Prevention (approval ID: IPD-2019-002, and IPD-2020-011).

### 2.4 Humoral immune responses induced by the FL46 vaccine

Serum samples were harvested from the mice 2 weeks and 8 months post-vaccination, and 8 weeks, 14 weeks, and 32 weeks post-infection. The FL46-specific antibodies were detected via indirect ELISA. Briefly, FL46 dissolved in 0.05 M carbonate buffer (0.5 μg/mL) was added to the 96-well plate and incubated at 4°C overnight. The plate was blocked with PBS containing 1% BSA at 37°C for 2 hours. The plate was incubated with diluted serum at 37°C for two hour. After washing five times with PBS containing 0.05% Tween-20 (PBST), the plate was incubated with HRP-conjugated goat-anti-mouse IgG, IgG1, IgG2a, or IgM at 37°C for two hour. After washing five times with PBST, the plate was incubated (15 min, RT) with TMB substrate solution. The reaction was stopped with 2M H2SO_4_, and the optical density (OD) at 450 nm was measured.

### 2.5 Cellular immune responses induced by the FL46 vaccine

Serum samples of mice were harvested. Samples were centrifuged at 4°C, and the supernatant was frozen at −80 °C. Ten cytokines (IL-1, TNF-α, IFN-γ, IL-2, IL-6, IL-4, IL-5, IL-10, KC, IL-12p70) were detected using the cytokine Liquid-phase chip of Luminex from LabEx (Shanghai, China). Briefly, 1 × beads were transferred to the assay plate and washed twice with Bio-Plex wash buffer. The plate was coated with the diluted standards, samples, and PBS, and incubated in the dark, shaking at 850 ± 50 rpm for one hour at room temperature. After washing three times, the detection antibody diluent was added and incubated for 30 minutes. Then, dilute SA-PE was added and incubated for 10 minutes at room temperature. After washing the plates three times, the assay buffer was added and incubated for 30 seconds. The plates were read with instructions for the Bio-Plex System.

### 2.6 Reverse transcription–quantitative PCR

Total RNA from mice livers was extracted with Trizol. After the concentration and purity were detected, RNA was reverse transcribed into complementary DNA (cDNA) using a reverse transcription kit (EnzyArtisan Biotech, Shanghai, China). RT- qPCR was used to detect the expression of collagen I and III genes using Fast SYBR Green master Mix (Bio-Rad, Hercules, CA, USA). The primers of the genes are shown in **Table 1**.

**Table 1:** Primer sequences for the related genes.

Gene name	Primer sequence 5’-3’
*GAPDH*	F: CATCACTGCCACCCAGAAGACTG R: ATGCCATGAGCTTCCCGTTCAG
*Collagen I*	F: CCTCAGGGTATTGCTGGACAAC R: CAGAAGGACCTTGTTTGCCAGG
*Collagen III*	F: GACCAAAAGGTGATGCTGGACAG R: CAAGACCTCGTGCTCCAGTTAG

### 2.7 Western blotting

Tissues of the mouse liver were lysed using the radioimmunoprecipitation assay (RIPA) lysis buffer. The lysates were centrifuged at 12,000 × *g* for 10 min, and the supernatant was collected to determine protein concentration using the BCA method. 5 × loading buffer was added to the liver tissue lysate and mixed thoroughly. After being denatured in a metal bath at 100°C for 10 min, the samples were loaded into the wells of a sodium dodecyl sulfate (SDS)-polyacrylamide gel (Beyotime Biotechnology, China). SDS-polyacrylamide gel electrophoresis was performed at 90 V for approximately one hour. The proteins were transferred to polyvinylidene fluoride (PVDF) membranes. After blocking PVDF membranes, antibodies against α-SMA(CST, MA, USA), Collagen I(Bioss, Beijing, China)were added and incubated at 4°C overnight. And Goat anti-Rabbit-IgG(H+L)(CST, MA, USA)was added and incubated for one hour. After exposure and photography, we used the Image J software to analyze the gray values of the bands.

### 2.8 Flow cytometry

The part of the spleen was disrupted mechanically. Tissue suspension was passed through a 70μm cell strainer and washed with a complete DMEM medium to obtain a spleen single-cell suspension. Single cells were stained according to standard protocols. The proportions of T and B lymphocytes were detected with the following antibodies (BD Biosciences), including Fixable Viability Stain BV605, CD3-FITC, CD19-PE, and B220-FITC.

### 2.9 Histopathology

Liver tissue of mice at the same position was taken, and paraffin sections were made. The paraffin sections were stained with Potassium dichromate, iron hematoxylin, ponceau, phosphomolybdic acid, and aniline blue by dewaxing to water. After staining, it was dehydrated with anhydrous ethanol, transparent with xylene, and sealed with neutral gum. Microscopic examination revealed that the collagen fibers appeared blue. The area of collagen fibers was detected to evaluate the extent of liver fibrosis.

### 2.10 Statistical analysis

Data were analyzed with GraphPad Prism Version 10.3. We assessed differences between groups using parametric analysis, including one-way or two-way analysis of variance, and Student’s T-test. The data were presented as the means ± standard deviation. The test criterion was P < 0.05.

## 3 Results

### 3.1 The epitope characteristics derived from *E. granulosus*

**Figure 1:**
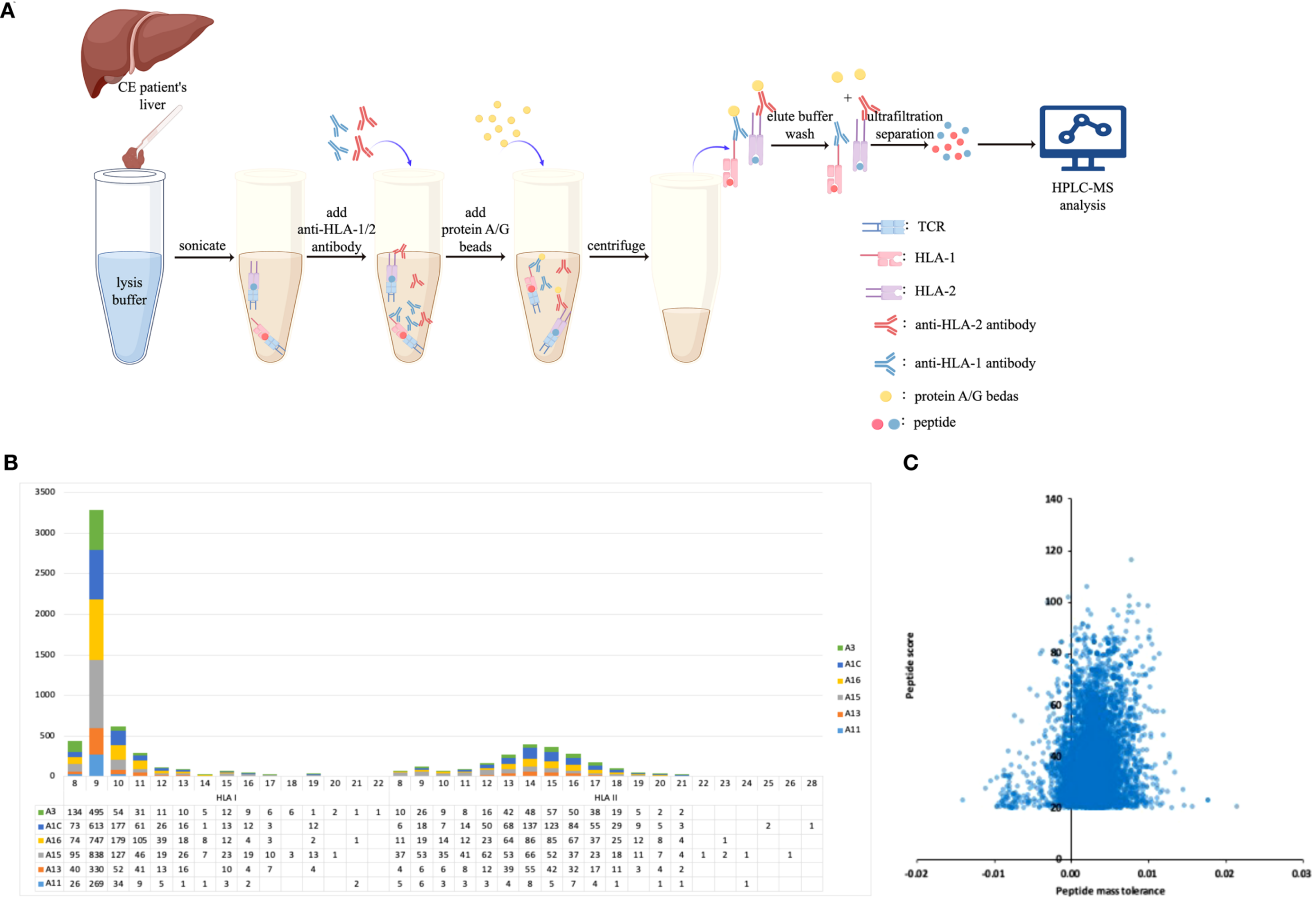
The screening of T-cell epitopes of *E. granulosus*. **(A)** The strategy of antigenic epitope screening (draw with Figdraw software); **(B)** The length distribution map of identified peptides; **(C)** The quality distribution map for the identified peptides.

HLA-bound epitopes of *E. granulosus* were screened from the livers of echinococcosis patients by the co-immunoprecipitation technique (**Figure 1A**). The length of peptides in the samples was basically concentrated on 8–11 amino acids (peptides binding with HLA class I molecules) and 13–17 amino acids (peptides binding with HLA class II molecules) (**Figure 1B**). We screened out 73 HLA class I molecular-binding peptides and 47 HLA class II molecular-binding peptides of *E. granulosus*. The quality errors of the identified peptide were mostly less than 0.01Da, which was in line with the high-precision characteristics of orbital trap mass spectrometry, indicating that the mass accuracy of the mass spectrometer was normal. The score of the spectrum-matching peptide (characterizing the credibility of peptide identification) is negatively correlated with the distribution of quality deviation. The higher the score is, the smaller the quality deviation is (**Figure 1C**).

We identified three epitopes of P1, P2, and P3 from *E. granulosus*. P1 bound with HLA class II molecules was shared by six samples. P2 and P3, bound with HLA class II and I, were shared by three samples. The synthesized single-epitope P1, P2, and P3 were analyzed by high-performance liquid chromatography (HPLC) and mass spectrometry. The results of HPLC showed that P1, P2, and P3 presented polypeptide peaks at 10.52 min, 8.59 min, and 7.26 min, and purities were 99.3%, 98.8%, and 99.7%, respectively. The molecular weights of P1, P2, and P3 analyzed by MS were 965.1 g/mol, 1037.1g/mol, and 997.1 g/mol.

### 3.2 Preparation and toxicity of FL46

**Figure 2:**
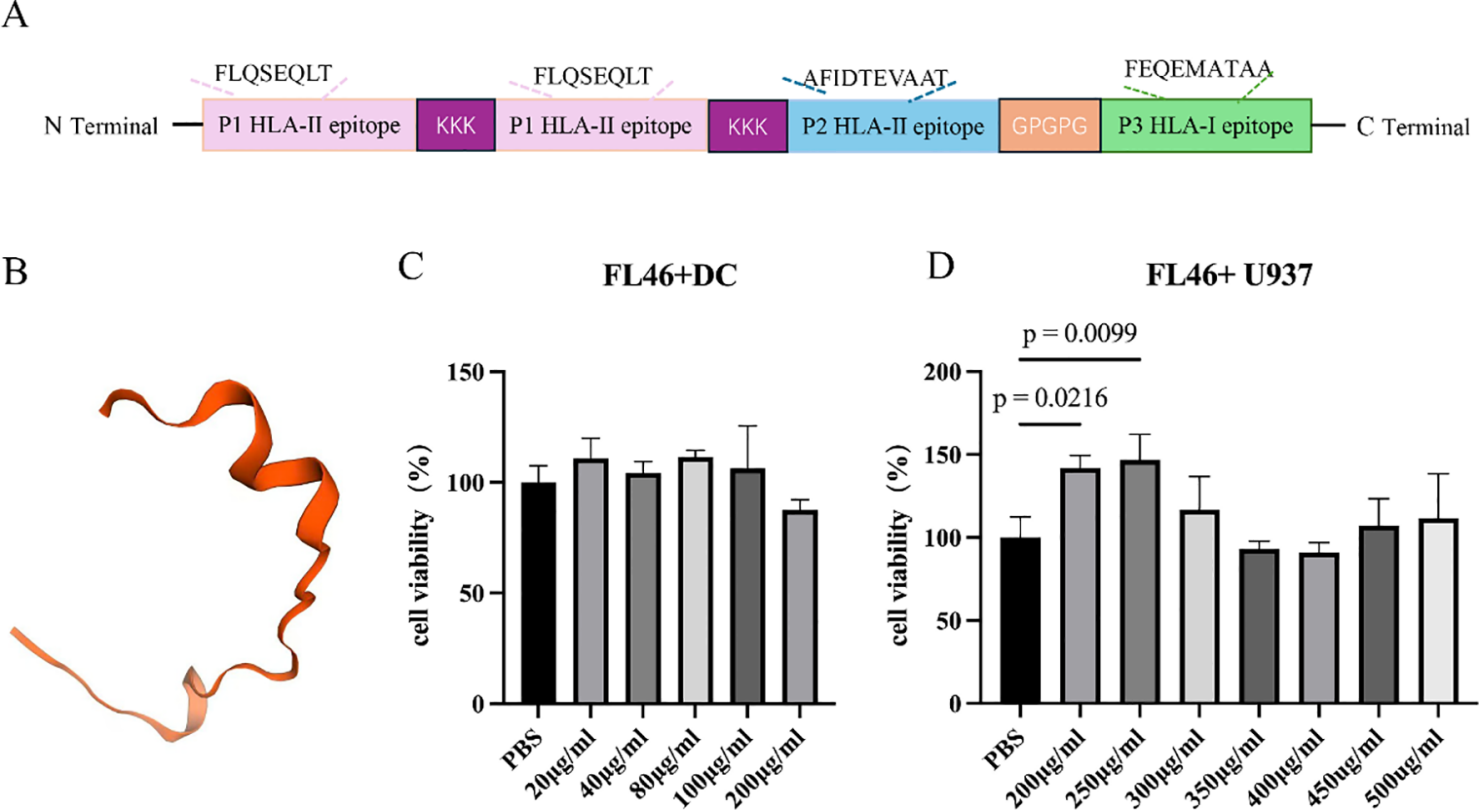
Preparation and toxicity of FL46. **(A)** Schematic diagram of FL46 construction; **(B)** The simulated 3D structure of FL46; **(C)** The detection of BMDCs’ proliferation; **(D)** The detection of U937’s proliferation.

The T-cell epitopes P1, P2, and P3 of *E. granulosum* were connected with appropriate linkers (KKK and GPGPG). The KKK linker was used to link the P1 and P2 epitopes (binding with HLA-II molecules), and the GPGPG linker was used to link the P2 (binding with HLA-II molecules) and P3 (binding with HLA-I molecules) epitopes. FL46 polypeptide of *E. granulosus* was constructed with P1, P2, and P3 (**Figure 2A**). The 3D structure of FL46 was simulated on the SWISS-MODEL website, which showed that FL46 presented a flexible band structure, and the spatial conformations of epitopes were not affected by the connection (**Figure 2B**). The result of HPLC showed that FL46 presented a peptide peak at 6.31min, with a purity of 96.9%, and the molecular weight of the FL46 analyzed by MS was 5044.8g/mol.

The proliferation of primary BMDCs and mononuclear cell lines (U937 cells) stimulated by FL46 was detected using CCK8 to evaluate the toxicity of FL46. Compared with the negative control, there was no significant difference in cell viability when primary BMDCs were stimulated with FL46 at different concentrations (20, 40, 80, 100, 200 μg/mL) for 24 hours (P > 0.05). Similarly, after U937 cells were stimulated with FL46 at concentrations (300, 350, 400, 450, and 500 μg/mL) for 24 hours, there was no significant difference in cell proliferation between the U937 and control group (P > 0.05). The results showed that FL46 at concentrations below 200 μg/mL has no effect on the viability of BMDCs (**Figure 2C**), and concentrations below 500 μg/mL did not inhibit the proliferation of U937 cells (**Figure 2D**).

### 3.3 Cellular immune responses induced by FL46

**Figure 3:**
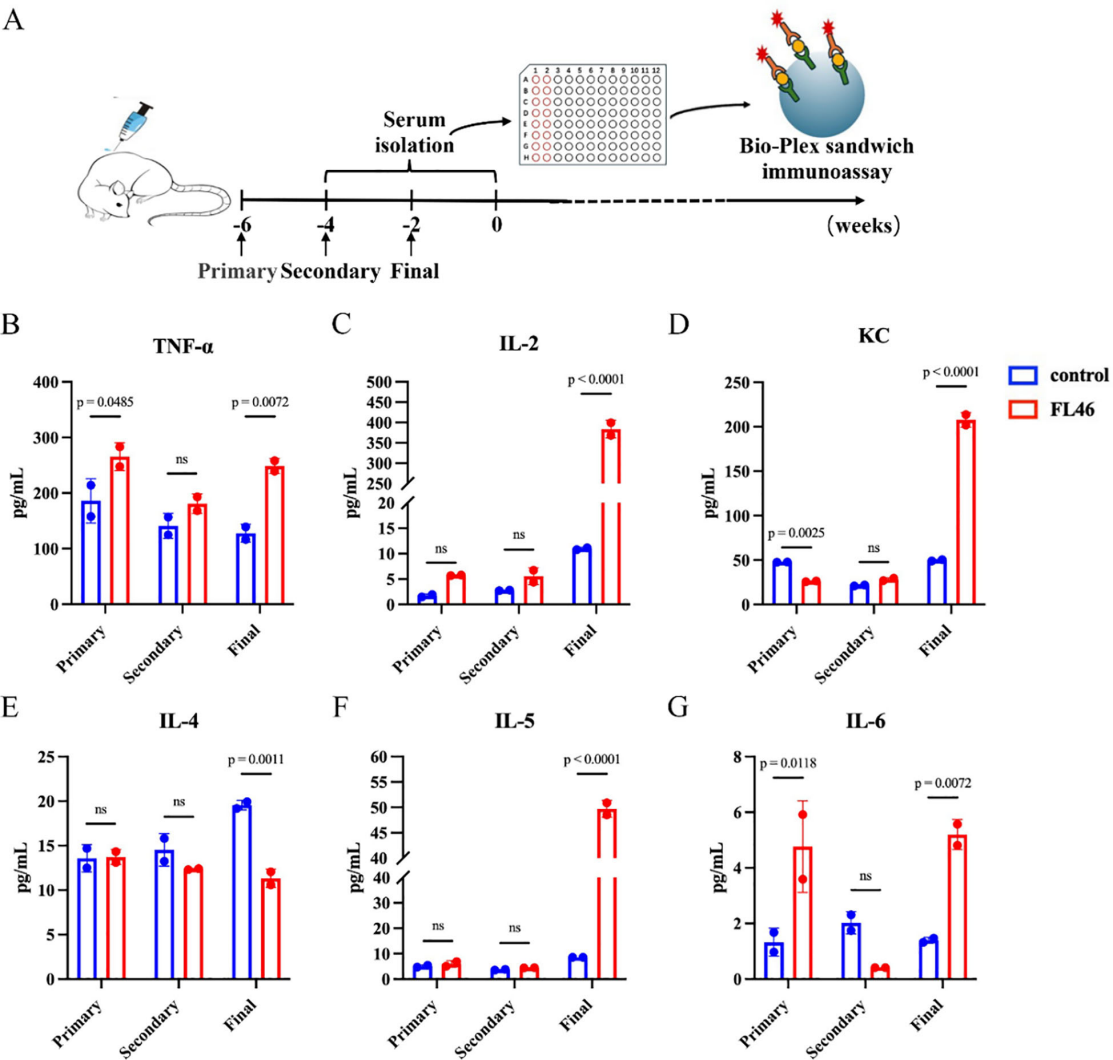
Cellular immune response induced by FL46 vaccine in C57BL/6 mice two weeks post-vaccination. **(A)** Vaccination strategy of FL46. **(B)** The expression of TNF-α in the serum of mice. **(C)** The expression of IL-2 in the serum of mice. **(D)** The expression of KC in the serum of mice. **(E)** The expression of IL-4 in the serum of mice. **(F)** The expression of IL-5 in the serum of mice. **(G)** The expression of IL-6 in the serum of mice.

The strategy of the FL46 vaccination is shown in **Figure 3A**. Two weeks after the third immunization with the mixture of FL46 and Freund’s adjuvant, the levels of cytokines such as TNF-α, IL-2, KC, IL-5, and IL-6 in the serum of mice increased (**Figures 3B-G**). The levels of IFN-γ, IL-5, and IL-10 increased in the vaccine group after being infected with protoscoleces (**Supplementary Figure S1**).

**Figure 4:**
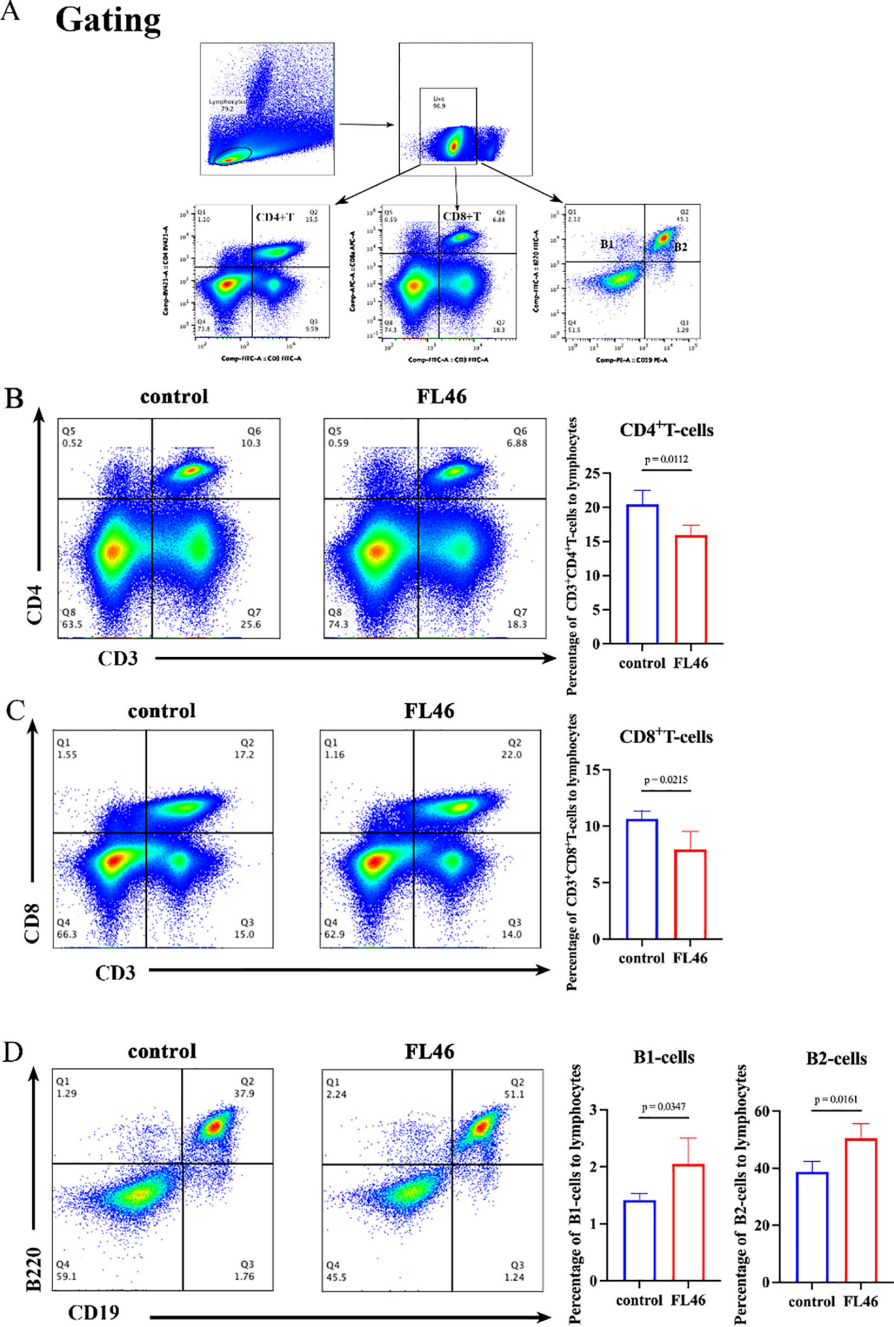
The changes in lymphocyte proportion induced by FL46 vaccine eight months post-vaccination in the spleen. **(A)** The gating strategy of splenic lymphocytes. **(B)** The changes in the proportion of CD4^+^T lymphocytes. **(C)** The changes in the proportion of CD8^+^T lymphocytes. **(D)** The changes in the proportion of B lymphocytes.

Flow cytometry results showed that eight months after the third immunization, the proportions of B1 and B2 in the splenic lymphocytes in the vaccine group were significantly higher than those in the control group (**Figure 4D**). In contrast, the proportion of T cells in the total lymphocytes was considerably lower (**Figures 4B, C**). The decrease in T cell proportion may be related to the increase in the proportion of B cells.

### 3.4 Humoral immune responses induced by FL46

**Figure 5:**
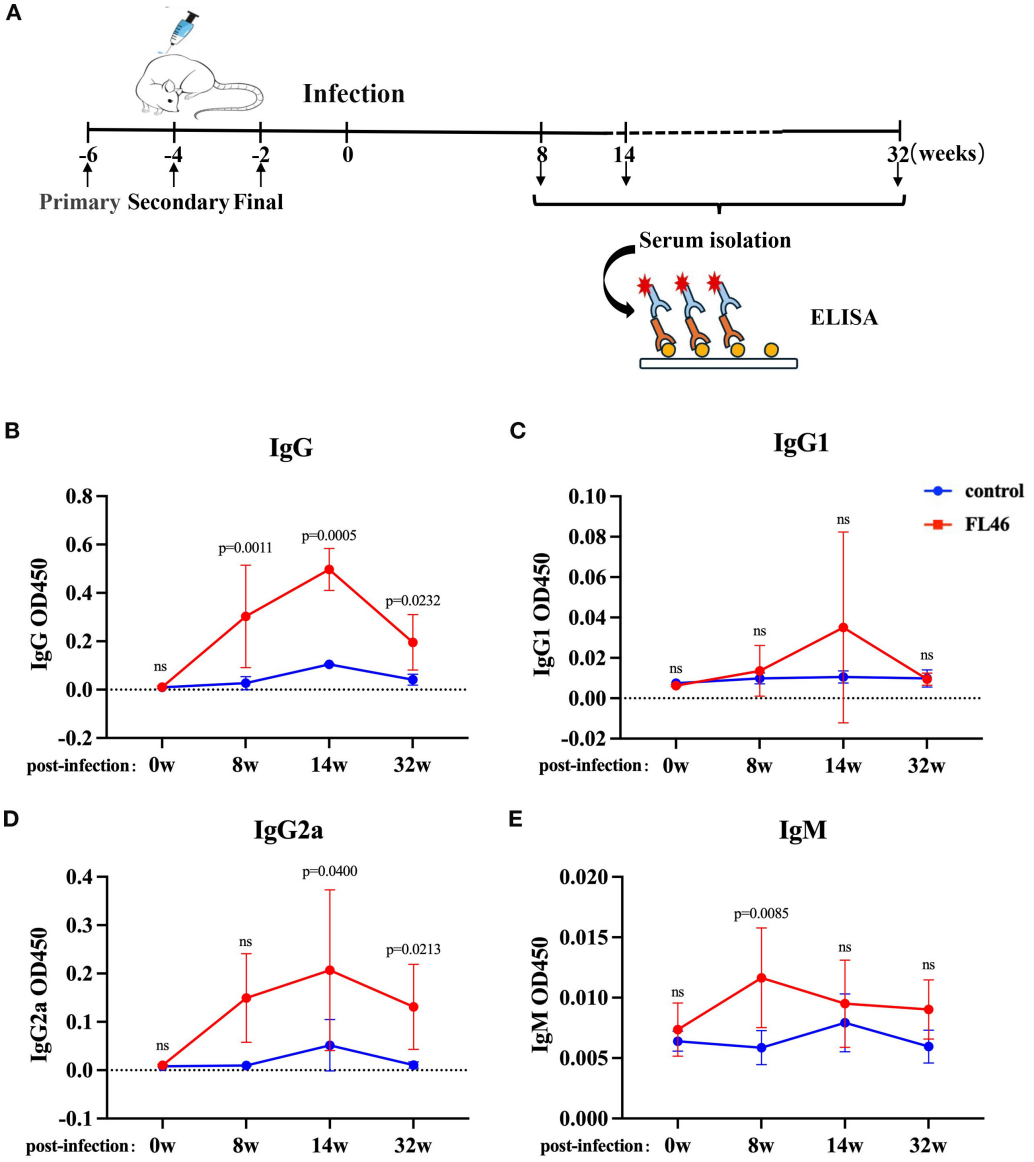
Humoral immune response induced by the FL46 vaccine in C57BL/6 mice after infection. **(A)** The timeline of vaccination and infection in mice; **(B)** Dynamic changes of IgG in the serum of mice; **(C)** Dynamic changes of IgG1 in the serum of mice; **(D)** Dynamic changes of IgG2a in the serum of mice; **(E)** Dynamic changes of IgM in the serum of mice. Ns represents no significant difference.

Healthy C57BL/c mice were subcutaneously injected with vaccines three times. The immunization and infection schedule was shown in **Figure 5A** to detect FL46- specific antibodies. Eight months after the third immunization, total IgG and IgG2a levels in the serum of mice significantly increased in the vaccine group. The level of IgM in the vaccine group peaked at eight weeks post-infection (**Figure 5E**). The level of IgG in the vaccine group increased significantly from eight to 32 weeks post-infection, and the IgG2a level increased from 14 to 32 weeks post-infection (**Figures 5B, D**). The levels of IgG and IgG2a at 32 weeks were lower than those at 14 weeks after infection, but still higher than those in the control group mice. There was no significant difference in the IgG1 level between the vaccine and control groups (**Figure 5C**). The results showed that FL46 could induce a strong humoral immune response. High levels of antibodies could last for at least eight months (**Supplementary Figure S2**). The humoral immune response induced by FL46 tends to Th1-type immunity.

### 3.5 Evaluation of the protective effect of FL46 against *E. granulosus*

**Figure 6:**
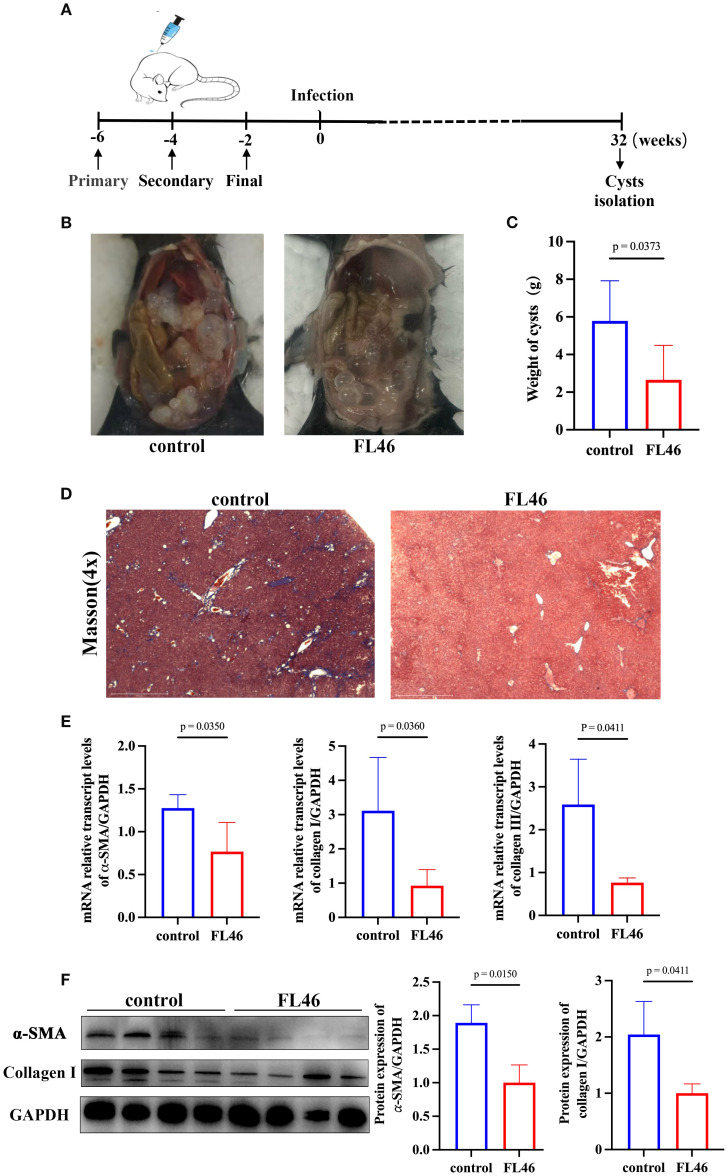
Evaluation of the protective effect of FL46 against *E. granulosus.* **(A)** The timeline of vaccination and infection in mice. **(B)** The size and number of abdominal cysts in mice. **(C)** The weights of abdominal cysts in mice. **(D)** The collagen area in the liver of infected mice. **(E)** Bar chart of α-SMA, collagen I, and III mRNA expression in mouse liver tissue. **(F)** The protein expression of α-SMA and Collagen I in the liver of infected mice.

Two weeks after the third immunization, protoscoleces were intraperitoneally injected into the mice (**Figure 6A**). The number and size of abdominal cysts in mice of the vaccine group were significantly smaller than those in the control group (**Figure 6B**). The cysts were removed and weighed. The weight of the cysts from mice of the vaccine group was 1.89 ± 1.83 g, and that from the control group was 4.64 ± 2.45 g. The rate of suppression cyst was 59.16% (**Figure 6C**). The results showed that FL46 could alleviate the infection of *E. granulosus*.

The area of collagen fibers in the liver of mice in the vaccine group was significantly smaller than that of mice in the control group (**Figure 6D**). The mRNA levels of α-SMA, collagen I, and III in the liver of mice from the vaccine group were significantly lower than those from the control group (**Figure 6E**). The protein levels of α-SMA and collagen I in the liver of mice from the vaccine group were significantly lower than those from the control group (**Figure 6F**). It was suggested that immunization with FL46 vaccines could reduce the worm burden and alleviate the degree of liver tissue fibrosis in mice infected with protoscoleces.

## 4 Discussion

Echinococcosis is a zoonotic parasitic disease distributed globally, seriously threatening human health and the development of animal husbandry. China is the country with the highest incidence of echinococcosis (18). By the end of 2023, there were 25,362 cases of echinococcosis in China, among which cystic echinococcosis accounted for 62.61% (19). At present, the treatment drugs for echinococcosis are single. There is a lack of safe and efficient vaccines. The technical reserves for disease prevention and control are seriously insufficient. Vaccines can not only inhibit the growth of cysts and damage to liver tissue (20) but also reduce the number of parasites in the definitive host dog, inhibit the growth and development of tapeworms, and decrease the risk of disease transmission (21). Therefore, the development of vaccines is of great significance for preventing and controlling echinococcosis.

Echinococcosis vaccines are classified into traditional vaccines, recombinant protein vaccines, nucleic acid vaccines, and synthetic peptide vaccines. Traditional vaccines use crude antigens of *E. granulosus* and have significant differences among batches, which cannot be put into large-scale use. Genetic engineering vaccines are recombinant protein vaccines constructed by cloning antigen molecules into expression vectors. They are relatively easy to preserve and have a lower cost (22). The most effective echinococcosis vaccine is the commercialized Eg95 recombinant protein vaccine for sheep, with a protective effect of 90% or more. However, it is limited due to insufficient cellular immunity, a short protection period, and limited protection against novel mutant strains (11, 23). DNA vaccines such as Eg95, EgA31, and antigen B can induce long-term humoral and cellular immune responses but may cause side effects such as autoimmune diseases (24). Peptide vaccines have strong specificity and pose no safety risks, but their immunogenicity is relatively low. When used in combination with adjuvants, significant protective effects can be achieved. Xiaoyu An et al. expressed eight T and B cell epitopes with high immunity on the surface of Vero cells and constructed a nanovesicle vaccine, achieving significant protective effects (25).

T and B cell epitopes of targets, such as Eg95, Ag5, EmTSP-3, and EmTIP, were predicted with biological software and combined to prepare multi-epitope vaccines. The immunogenicity of polypeptide vaccines is evaluated through bioinformatics, *in vitro*, and *in vivo* experiments (26–28). Immunopeptidomics, the mass spectrometric identification of human-leukocyte-antigen (HLA)-bound peptides isolated from infected cells, has recently provided key insights into pathogen peptides that can serve as potential T cell epitopes (29, 30). In our experiment, 120 peptides that were specifically bound to HLA-I or HLA-II were screened out through the strategy of immunopeptidomics. Among them, six patients share the P1, and three patients share the P2 and P3 peptides. We connected the three single epitopes P1, P2, and P3 through the appropriate flexible linkers KKK and GPGPG to construct the complex polypeptide FL46. The spatial structure of each epitope could not be affected by the others (31). A multi-epitope vaccine can activate a broader range of T and B cells, generating a more comprehensive immune response and effectively inducing protection (25). FL46 is an artificially synthesized polypeptide. When the concentration of FL46 is lower than 500μg/mL (equivalent to 20 times the vaccine concentration in mice), FL46 stimulated BMDCs and U937 cells without inhibiting the proliferation response of the cells, indicating that FL46 was safe and non-toxic.

It is the key to preventing or controlling Echinococcosis of vaccine inducing the host to produce humoral immunity and Th1/Th2 cellular immunity (32). In this study, the P1, P2, and P3 epitopes contained in FL46 could bind to MHC molecules and be presented to T cells, which effectively induced humoral and cellular immune responses. Mice were immunized with the compound epitope FL46 and Freund’s adjuvant three times. Eight months after the last immunization, both IgG and IgG2a titers in the serum of mice in the vaccine group were significantly higher than those in the control group. After being infected with protoscoleces, the titers of IgG and IgG2a in the vaccine group increased dramatically at eight weeks and peaked at 14 weeks. High levels of antibodies could last for at least eight months. The level of IgG2a was significantly higher than that of IgG1. Spleen B lymphocytes include B1 (CD19^+^B220^-^) and B2 (CD19^+^B220^+^), as well as immature and mature B cells. B1 lymphocytes are associated with the production of low-affinity antibodies, including IgM, IgA, low-affinity IgG, and IgE. B2 lymphocytes are related to the production of high-affinity antibodies, such as IgG and IgG2a (33). After immunization with FL46 for eight months, the proportion of B1 and B2 lymphocytes in the spleen increased, and B2 increased more significantly. It was consistent with the high levels of IgG and IgG2a in the serum of mice after immunization for eight months.

In human CE, a cytokine-related Th2 response leads to susceptibility to echinococcosis, but a Th1 response results in protective immunity against the disease (34). Another study also found that the Th1 immune response is more efficient than the Th2 one in controlling parasitic infection (35). In our study, two weeks after the last immunization, the levels of Th1-type cytokines TNF-α, IL-2, and Th2-type cytokines IL-5 and IL-6 in the serum of mice increased significantly. Among them, the levels of TNF-α and IL-2 cytokines were considerably higher than those of IL-5 and IL-6, and the response was more biased towards Th1-type. After the first immunization with polypeptide FL46 and Freund’s adjuvant, the level of IL-2 in serum increased significantly. After the third immunization, the level of IL-2 increased more significantly. IL-2 could stimulate T cells′ growth and differentiation, promote CTL′s the function, activate NK cells and macrophages, and induce significant cellular immune responses (36, 37).

The peptide vaccines have a relatively small molecular weight and weak immunogenicity. Therefore, it is necessary to enhance the immunogenicity of peptides by combining them with adjuvants (38). Freund’s adjuvants are classic emulsion adjuvants, including complete Freund’s adjuvant and incomplete Freund’s adjuvant. Complete Freund’s adjuvant can keep the vaccine at the injection site for a long time, prevent the degradation of polypeptides, and maintain the long-term immune response induced by the vaccine (39, 40). While enhancing the immune effect, Freund’s adjuvant causes inflammatory responses and aseptic abscesses at the injection site, which limits the application of Freund’s adjuvant in humans (41). For epitopes of FL46 combined with HLAI and II of human, it is our limitation of research to select Freund’s adjuvant compared with FL46. In the future, we will use clinically relevant adjuvants (e.g., Aluminum, TLR agonists, etc.) or mouse/human commonly used self-adjuvant components (e.g. PADRE, β-defensin, etc.) to enhance the immune efficacy of the vaccine.

There are significant differences in MHC molecules between humans and mice. The vaccine-induced protective effects in humans may not necessarily induce sound protective effects in mice (42). FL46 is a multi-epitope peptide screened out from the liver tissues of patients with echinococcosis. We evaluated the protective effect of FL46 using C57BL/6 mice, achieving a 59.16% reduction in cyst burden. It may be a certain degree of binding site conservation with the peptide in MHC in mice and HLA in humans. Ruibal P et al. showed that Qa-1b of mice is the functional homolog of HLA-E and shows similar peptide binding specificity, likely due to the high structural conservation of the peptide binding groove (43). Another possibility is that FL46 is a hapten that is not restricted by MHC molecules. When mixed with Freund’s adjuvant, it becomes a complete antigen, effectively inducing the host’s immune response.

The vaccine evaluation system is another limitation of our experiment. If we adopted primates as the evaluation system, the protective effect of FL46 may be evaluated more truly and effectively. Therefore, optimizing the vaccine evaluation system is also crucial for assessing the protective effect of human vaccines. In recent years, organoids or transgenic mice have mainly been used to evaluate the protective effects of human vaccines (44).

## 5 Conclusion

In this study, we screened out the HLA-bound epitopes (P1, P2, and P3) of *E. granulosus* from the liver tissues of echinococcosis patients using the strategy of co-immunoprecipitation combined with mass spectrometry. The flexible couplers KKK and GPGPG were used to connect P1, P2, and P3 to synthesize the complex epitope vaccine FL46. FL46 is convenient to produce, safe, and non-toxic. C57BL/6 mice were immunized with Freund’s adjuvant three times, which could induce a 59.16% cyst reduction rate. FL46 could induce a robust immune response with a bias towards a Th1-type phenotype. The high level of antibodies could last for at least 8 months. Our research showed that FL46 is a highly promising multi-epitope vaccine against echinococcosis.

## Data Availability

The original contributions presented in the study are included in the article/**Supplementary Material**. Further inquiries can be directed to the corresponding author.

## Supplementary material

Supplementary Figure 1 The level of cytokines in the serum of mice after vaccination and infection. (A). The level of TNF-α. **(B)** The level of IFN-*y*. **(C)** The level of IL-1β. **(D)** The level of IL-2. **(E)** The level of IL-5. **(F)** The level of IL-10. **(G)** The level of IL-12P70. -6w represents six weeks before infection (before immunization); 0w represents the timepoint of infection (two weeks after three immunizations); 8w represents eight weeks after infection; 14w represents 14 weeks after infection.

Supplementary Figure 2 The changes in antibody levels eight months post-vaccination. **(A)** The level of IgG in the serum. **(B)** The level of IgG1 in the serum. **(C)** The level of IgG2a in the serum. **(D)** The level of IgM in the serum.
