# Supplementary figures and images for: Development and protective efficacy of multi-epitope vaccine FL46 against cystic echinococcosis
Source: Front Immunol. 2025 Oct 16;16:1686959. doi: 10.3389/fimmu.2025.1686959 (PMC12571753; doi:10.3389/fimmu.2025.1686959)

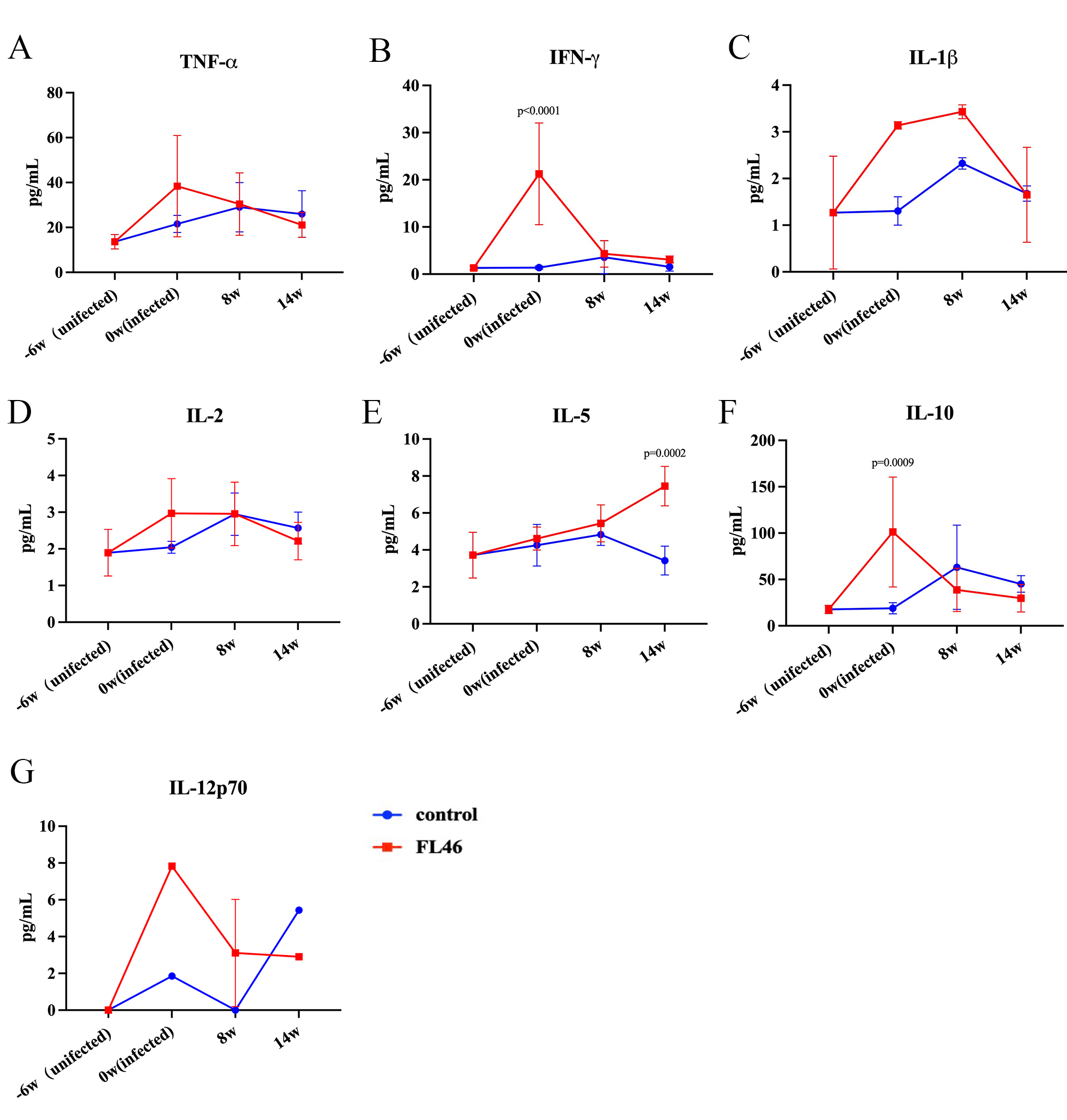

Supplement: Supplementary Figure 1 — The level of cytokines in the serum of mice after vaccination and infection. (A). The level of TNF-α. (B) The level of IFN-y. (C) The level of IL-1β. (D) The level of IL-2. (E) The level of IL-5. (F) The level of IL-10. (G) The level of IL-12P70. -6w represents six weeks before infection (before immunization); 0w represents the timepoint of infection (two weeks after three immunizations); 8w represents eight weeks after infection; 14w represents 14 weeks after infection. [file Image1.tif]

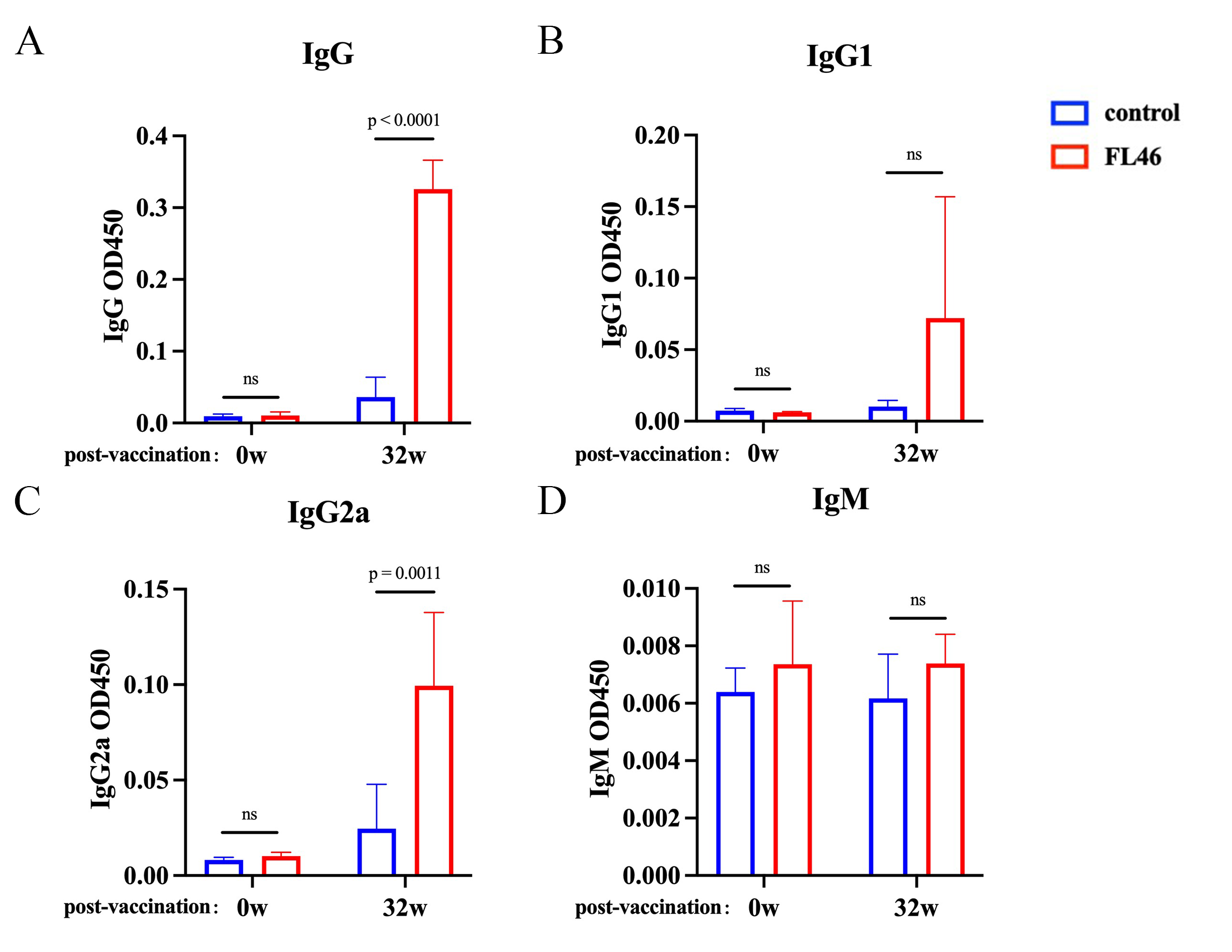

Supplement: Supplementary Figure 2 — The changes in antibody levels eight months post-vaccination. (A) The level of IgG in the serum. (B) The level of IgG1 in the serum. (C) The level of IgG2a in the serum. (D) The level of IgM in the serum. [file Image2.tif]
